# Climate Change and Pediatric Skin Health: Emerging Threats, Innovations, and Equity Gaps

**DOI:** 10.7759/cureus.91397

**Published:** 2025-09-01

**Authors:** Harleen K Multani, Kiratpreet K Sraa, Hana Abbas, Shikha Gandhi, Lauryn J San Juan, Elaine H Pan, Fiona Riedel, Saira Khan

**Affiliations:** 1 Dermatology, Meharry Medical College School of Medicine, Nashville, USA; 2 Dermatology, Arizona College of Osteopathic Medicine, Glendale, USA; 3 Orthopedic Surgery, Meharry Medical College School of Medicine, Nashville, USA; 4 Dermatology, Marian University Wood College of Osteopathic Medicine, Indianapolis, USA; 5 Dermatology, University of South Florida, Tampa, USA; 6 Dermatology, Cleveland State University, Cleveland, USA; 7 Pediatrics, Baylor College of Medicine, Houston, USA

**Keywords:** atopic dermatitis, climate change and health, pediatric dermatology, pediatric skin changes, skin barrier dysfunction

## Abstract

Climate change poses unique dermatologic risks to children due to immature skin barrier function, weakened immune systems, and dependence on caregivers. Under stable environmental conditions, pediatric skin maintains homeostasis through balanced barrier function, microbiome diversity, and immune regulation. The changing climate disrupts these protective mechanisms through rising temperatures, air pollution, ultraviolet (UV) radiation, and adverse weather conditions. Research demonstrates these environmental stressors exacerbate atopic dermatitis (AD), infectious dermatoses, and infestations, all while disproportionately affecting marginalized communities. Current clinical approaches often fail to address the climate-related dimensions of pediatric skin disease, relying on traditional therapies without environmental adaptation. Emerging solutions, such as climate-resilient skincare formulations, teledermatology, and community-based interventions, show promise for more effective management. This review examines the pathophysiological effects of climate change on pediatric skin, evaluates current and emerging care strategies, and identifies critical gaps in the literature. Challenges include limited pediatric-specific climate research, healthcare disparities in vulnerable populations, and inadequate integration of dermatologic concerns into climate policy. Despite these barriers, advances in preventive dermatology and community-based interventions offer opportunities to improve outcomes. Future progress will depend on interdisciplinary efforts to develop climate-adaptive skin care frameworks that protect children's health in a dynamic world.

## Introduction and background

Climate change is increasingly recognized as a public health threat, promoting a wide variety of diseases by both direct and indirect pathways. The pediatric population, especially neonates and infants, is subject to increased skin susceptibility due to developmental differences in barrier structure and function. The stratum corneum of infants is 30% thinner than that of adults and is characterized by increased disorganization in addition to cell turnover [1]. As a result, even minimal exposure to environmental stressors can lead to adverse dermatological reactions in young children. The different indicators of climate change both directly and indirectly affect pediatric skin health. As the global mean temperature increases, heat-related disease has also increased. Children’s poorly developed thermoregulatory mechanisms leave them susceptible to heat-related illnesses [2]. Their dependence on caregivers, inability to reapply sunscreen, and struggle to adjust protective sun clothing independently have a disproportionate impact on their skin. Extreme weather is likely to lead to overcrowding as a result of population displacement with poor sanitation, thereby facilitating the spread of infestations and infections, in which children are most vulnerable [2]. Not only may increased temperatures correspond to more time spent outdoors without protective clothing in children, but it is also suggested that skin cancer risk is higher with the same ultraviolet (UV) dose [3]. Pediatric skin health is particularly affected by climate change and its associated warming. Despite the evidence, there are still gaps in the literature with respect to long-term outcomes, preventative measures, and intervention efficacy in one of the most at-risk populations.

## Review

Pathophysiology

Children are disproportionately susceptible to the dermatologic effects of climate change, particularly those living in impoverished areas [4]. Their developing physiology, immature immune responses, and dependence on adults for care make them disproportionate bearers of climate change, attributed to adverse effects. Climate-change stressors such as rising temperatures, pollution, and UV radiation (UVR) disrupt their skin equilibrium. For instance, young children have an underdeveloped sweat response and a higher surface area to mass ratio, limiting their ability to dissipate heat efficiently during times of thermal stress [5]. Additionally, this makes them less efficient at lowering their core temperature if overheated. It has been demonstrated that children not only generally produce less sweat but also require a higher core temperature at which sweating can begin [6]. This physiologic immaturity increases their risk for heat-related dermatoses such as miliaria, a heat rash that presents secondary to sweat gland occlusion, and exacerbates preexisting inflammatory conditions such as atopic dermatitis (AD) [7,2].

UVR, amplified by ozone depletion, introduces both acute and chronic cutaneous risks, especially in pediatric populations. Of particular note, increasing UVB exposure, poor sunscreen habits, and greater time spent outdoors increase sunburn likelihood in pediatric populations [5]. Beyond these immediate effects, chronic UV exposure during early life increases the risk of photoaging and skin cancers, including melanoma and basal cell carcinoma [8]. Complicating this, UVB exposure has been found to impair sweat gland function and reduce nitric oxide-mediated vasodilation, which is critical for thermoregulation in children [5]. Additionally, darker-skinned children, Fitzpatrick type IV-VI, may present with post-inflammatory hyperpigmentation rather than erythema following prolonged UV exposure, which can delay diagnosis and mask the extent of UV damage [8]. 

Air pollution is another major climate-exacerbated stressor that has dermatologic implications. Particulate matter (PMx) and polycyclic aromatic hydrocarbons (PAHs) can penetrate the skin barrier and induce oxidative stress responses. These pollutants are found to exacerbate conditions such as AD by increasing the production of inflammatory cytokines and impairing key proteins such as filaggrin in the skin barrier. Studies have also found an increased risk of AD in children who live closer to main roads, implicating traffic-related pollution in the development of AD [9]. Notably, long-term exposure to air pollution has also been found to be associated with vascular dysfunction and signs of endothelial damage in children, which raises concerns for how these effects may hinder cutaneous microvascular function and thermoregulation [5]. 

Extreme weather events such as floods and heat waves exert both direct and indirect dermatologic impacts on pediatric populations. Essentially, dermatologic diseases are likely to spike after flooding events, as they can lead to prolonged exposure to contaminated water [10]. Subsequently, an increase in skin trauma, secondary bacterial infections (e.g., *Vibrio vulnificus*, *Pseudomonas aeruginosa*, Methicillin-resistant *Staphylococcus aureus* (MRSA)), and irritant contact dermatitis due to sewage and chemical runoff is reported [10]. Standing water also creates an environment that is optimal for fungal overgrowth, which only poses additional risk for children who have compromised skin barriers or limited hygiene access. Extreme heat events, attributable to global warming, have also seen an upsurge and foster heat-exacerbated skin diseases such as Grover’s disease, folliculitis, and intertrigo [10]. Importantly, children are disproportionately affected by these climate-related exposures due to increased vulnerability and lack of agency [10]. 

Psychosocial stress, including climate anxiety, can also exacerbate pediatric skin diseases through multiple neuroimmunologic pathways, as summarized in Table 1. Stress activates the hypothalamic-pituitary-adrenal (HPA) axis, triggering the release of glucocorticoids, which modulate skin inflammation and barrier function. The skin expresses peripheral stress responses, enabling local production and response to corticotropin-releasing hormone (CRH) in keratinocytes, melanocytes, and mast cells [11]. Chronic stress can dampen the responsiveness of the central HPA axis while enhancing local Th2-mediated inflammation, particularly in AD, due to impaired glucocorticoid feedback and mast cell degranulation. CRH also increases the release of pro-inflammatory cytokines and vascular permeability, which contributes to flares of stress-responsive skin conditions such as psoriasis, alopecia areata, and chronic urticaria. Early-life stress has also been shown to alter HPA axis activity and Th1/Th2 cytokine profiles, increasing long-term susceptibility to inflammatory skin diseases [11].

**Table 1:** Mechanisms linking psychosocial stress and pediatric skin disease HPA: hypothalamic-pituitary-adrenal; CRH: corticotropin-releasing hormone; IL: interleukin; TNF-α: tumor necrosis factor-alpha Adapted from Zhang et al. [11].

Stress Mechanism	Cellular and Molecular Mediators	Pathophysiological Outcome	Associated Dermatological Condition
Systemic HPA axis activation	Hypothalamus, pituitary, adrenal glands, glucocorticoids	Modulates systemic skin inflammation and impairs barrier function	Atopic dermatitis, psoriasis
Peripheral skin stress response	Keratinocytes, melanocytes, mast cells, CRH	Local production of stress hormones and inflammatory mediators	Alopecia areata, vitiligo, urticaria
Immune polarization	Th2 cells, mast cells, cytokines	Enhanced local Th2-mediated inflammation and mast cell degranulation	Atopic dermatitis
Vascular and inflammatory signaling	Pro-inflammatory cytokines (e.g., IL-1, TNF-α), CRH	Increased vascular permeability and flare of inflammatory lesions	Psoriasis, chronic urticaria
Early-life stress	HPA axis, Th1/Th2 cytokine profiles	Long-term dysregulation of immune response and stress reactivity	Increased susceptibility to inflammatory skin diseases

Innovations in prevention and care

Climate change has introduced new dermatologic challenges in pediatrics, demanding innovative strategies in prevention and treatment that address both biologic vulnerability and structural inequities. One of the most promising developments in pediatric dermatologic prevention is the integration of wearable UVR monitoring devices. In a field study, adolescents at an outdoor event who wore UV sensors demonstrated significantly improved sunscreen and sunglasses use compared to baseline behaviors [12]. This real-time feedback system acted as a behavior prompt, allowing adolescents, a traditionally hard-to-reach population, to engage in sun-protective behaviors at periods of high-risk UV exposure. The repercussions are particularly applicable in the climate change era, in which increased UV index variability heightens pediatric skin damage risk. Building on this model, another study measured parents’ and children’s acceptability of UVR sensors and reported high satisfaction and increased awareness of sun exposure in users [13]. Notably, over 60% of children used the device consistently when going outdoors, showing strong adherence even in pediatric populations. Collectively, these findings demonstrate the feasibility of integrating wearable technologies into public health initiatives. 

Technological innovation in pediatric teledermatology, particularly in AI-amplified technology, has also enhanced access to pediatric dermatological care. Pediatric skin ailments such as rashes, AD, and infections saw an increase in rates of up to 70% to 89% concordance between child virtual consultations and traditional visits [14]. This finding is especially relevant against the backdrop of the global scarcity of child dermatologists, a problem that disproportionately affects low-resource and climate-exposed communities. Deployment of AI to maximize diagnostic precision in models of asynchronous consultation also reduces reliance on specialist time availability, promoting equality of access to dermatologic care in the face of rising climate-related skin health demand.

Apart from diagnostics and surveillance, innovation in skincare formulation is gaining traction as a strategy for maintaining pediatric skin homeostasis. Climatic stressors such as raised temperature, air pollution, and humidity variation have the potential to destabilize the skin barrier, especially in pediatric cohorts whose immune system and epidermal defenses have not fully matured. Emerging evidence supports the use of microbiome-stabilizing emollients and thermally stable sunscreens to reverse these adverse environmental effects [14]. While pediatric-specific products are still few, this direction of research aligns with the broader call for climate-resilient skincare that is responsive to children's unique physiologic profiles.

Along with individual-level interventions, policy-level interventions are central to moving skin health equity forward. Schools represent a critical point of intervention due to the high amount of time children spend outdoors during school hours. Guy et al. pointed out that school-based interventions, from environmental changes like shaded areas to sun-safety education, are not only effective but also underutilized [15]. They discovered that fewer than half of U.S. schools allowed students to apply sunscreen throughout the day, and even fewer had policies encouraging protective clothing or decreasing peak-hour sun exposure. These gaps in implementation reveal a discrepancy between clinical evidence and policy implementation, a discrepancy that must be resolved to maximize the use of schools as protective environments in the context of climate change. This finding assumes added importance in the context of global health planning. Sheffield and Landrigan argue that policy action by bodies like the World Health Organization must officially include dermatologic risks in broader climate health strategies [16]. Their review underscores that children under the age of five bear over 88% of the burden of climate change disease, yet dermatologic impacts are infrequently prioritized in global or regional adaptation agendas. Incorporating sun protection into school-based and community-level climate resilience policies would bridge this gap, such that skin health is not treated in a vacuum from other pediatric climate vulnerabilities.

Despite such progress, a number of challenges hinder the widespread deployment of such innovations. Cost is a significant barrier, as complex wearables and AI-powered platforms may be too costly in resource-constrained settings or not reimbursable under insurance. Furthermore, regulatory ambiguity around AI use in pediatrics, including concerns about data privacy and diagnostic accuracy, has slowed clinical uptake [14]. Above all, perhaps, the lack of child-focused research and commercial investment in child-oriented skincare and digital technologies generates a cycle of underdevelopment in this field. Without targeted investment and cross-sector collaboration, the full potential of these technologies will not be unlocked.

Community empowerment and low-cost strategies 

In disaster scenarios, having simplified clinical algorithms can streamline care and ensure that critical needs are consistently addressed. The Heat Tolerance Test (HTT) and Heat-related Illness Screening Tool (HIST) were developed to evaluate an individual's capacity to function in high temperatures and to identify risk factors contributing to heat intolerance [17]. These tools may be especially valuable for children, particularly athletes involved in outdoor sports such as track, cross country, cycling, soccer, and swimming, who may be more affected by climate change and heat-related illnesses, as HIST accounts for environmental, physical, social, and population-specific risk factors. Scoring systems like these allow physicians to triage and tailor education for vulnerable groups more effectively. Similarly, A CLIMATE, a clinical framework created by Nicholas et al., offers a structured, climate-conscious approach to patient care that begins with emergency stabilization and expands to include climate-focused history-taking, integrated treatment planning, and patient education [18]. While originally intended for the emergency department, this framework is easily adaptable to outpatient and inpatient dermatology settings. Given the psychological toll that climate-driven skin conditions can have on children, incorporating mental health screening tools into routine care is equally important. Commonly used tools such as the Children’s Global Assessment Scale, Strengths and Difficulties Questionnaire, and Revised Children’s Anxiety and Depression Scale can help identify psychosocial needs [19]. However, further consideration should be given to whether these tools assess alexithymia, which may hinder effective communication and limit the quality of care children receive if overlooked.

In addition to having simplified algorithms for consultation and screening, implementing one for skin barrier repair is equally essential. A climate-conscious skincare routine should include gentle cleansing and exfoliation, consistent moisturizing with barrier creams or emollients, application of topical antioxidants, and sun protection to combat UV damage [20]. Consistently applying each step can help address pollutant- and climate-driven skin issues from multiple angles. Antimicrobial peptides may also aid in restoring the skin’s tight junctions, decreasing itch, and suppressing inflammation, particularly in those with AD [21]. Pruritus is especially problematic in pediatric populations due to the limited ability to resist scratching, which can worsen the skin barrier and increase infection risk. In developing countries where resources may be limited, such as in Bangladesh, sunflower seed oil rich in linoleic acid may be a practical alternative, as it has demonstrated positive effects on the skin barrier when used on children [22]. To ensure broader implementation, algorithms should continue to consider culturally and economically viable alternatives such as oils derived from soybeans, cotton seeds, or corn. Additionally, future studies should evaluate the potential risks of excessive linoleic acid use and possible allergic responses.

When caring for children during stressful situations such as natural disasters, thorough yet simplified parent education is essential. One foundational recommendation is to encourage a Mediterranean diet rich in nutrients along with high water intake, which may aid in protecting against climate-aggravated skin conditions [20]. Additionally, guiding parents and caregivers through the simple four-step skincare routine mentioned above can help to counteract pollutant damage and reduce discomfort. If resources permit, dermatologists may want to advise parents to use micellar cleansing water as the first step in the child’s skincare routine, as a 2016 study conducted in Korea found that micellar cleansing water was 98% effective at removing PMx from the skin [23]. However, the specific brand of micellar cleansing water was not disclosed, and such products may not always be readily accessible in disaster scenarios. In these cases, parents should be advised to use a gentle, fragrance-free cleanser as an alternative, followed by application of a barrier cream and antioxidants such as vitamin C or vitamin E to improve the skin’s defense [24]. Prioritizing the restoration of the skin barrier and mitigating oxidative stress is fundamental to minimizing further skin damage.

If a child is already affected by dermatologic conditions, such as sunburns, skin cancer, AD, or acne, being outdoors without proper protection may lead to a vicious cycle of inflammation and worsening symptoms. Preventive strategies such as repeated sunscreen application, use of UPF clothing, and minimizing sun exposure are crucial to reduce further UV-induced damage. Counseling children and caregivers on the reasoning behind these practices may improve adherence, as individuals are more likely to follow recommendations when they understand the rationale behind them. Free, evidence-based sun safety curricula such as “Ray and the Sunbeatables,” “SunWise,” and “SunSmart U” were implemented at seven Nevada schools and revealed that the perceived importance of sun protection declined with age, with 60.2% of high school students believing that tans improved their appearance [25]. These findings underscore the need to target adolescents when promoting sun-protective behaviors. Introducing these programs at a younger age may also have a long-lasting impact, as they instill habits early on, are cost-friendly, and are easily accessible to both children and parents. While bleach and sodium bicarbonate baths or treatments involving bleach-soaked pajamas have been proposed to improve the skin microbiome, their efficacy remains controversial, with some studies showing no remarkable advantage over water baths alone [26-28]. These insights warrant further research to better understand the clinical benefit of bleach baths, especially when used for skin conditions aggravated by the effects of climate change.

In regions where climate-related disasters are increasingly common, proactive preparedness can greatly improve dermatologic outcomes, especially when resources are limited. A 2020 survey of 242 dermatologists found that only 28.9% had received disaster preparedness training, and just 16.4% felt comfortable managing nuclear or radiation-related injuries. Additionally, a substantial 74.7% agreed that formal disaster preparedness training should be incorporated into the dermatology curriculum [29]. Although the survey did not assess preparedness for pediatric care specifically, it highlights a critical gap in competency and emphasizes dermatologists’ desire to feel more prepared should these particular situations arise. With the increasing frequency of wildfires, hurricanes, and floods, it is vital to incorporate disaster-response training early in medical education and reinforce these skills throughout clinical practice to better equip clinicians and the communities they serve. Special emphasis should be placed on training programs in urban heat islands such as New York City, San Francisco, Houston, and New Orleans, where the risk of extreme heat and heat-related illnesses is higher, especially for children who are among the most vulnerable populations [30].

In addition to understanding local climate risks, physicians should also maintain broad disaster knowledge in case they choose to deploy to other regions in need. Incorporating realistic, high-stress simulations that include pediatric dermatologic care could promote an increased level of confidence and preparedness to serve in these settings. As part of a comprehensive response strategy, dermatology first-responder kits should include gentle cleansers, micellar cleansing water, topical antioxidants, sunscreen, barrier cream, and emollients. Institutions should consider stocking and distributing these items to displaced or affected children, especially when access to personal belongings is limited. Additionally, having pre-printed educational pamphlets on hand can aid in reinforcing instructions, reducing caregiver stress, and ultimately improving patient adherence to these protective measures. In addition, increased atmospheric carbon dioxide (CO₂) levels can diminish the nutritional content of food crops [2]. While this poses a health concern for the population at large, it may disproportionately impact children, who rely heavily on vitamins and nutrients to support healthy growth and development. To mitigate this risk, high-risk communities could consider implementing greenhouses as a means to grow reliable, nutrient-rich food sources in a controlled environment, specifically during climate-related disasters or when traditional crop yields are compromised. Incorporating AI into these systems may further enhance their effectiveness, as it has demonstrated 98% accuracy in detecting pests, monitoring weather fluctuations, and identifying defective crops to improve efficient production [31]. Leveraging this technology within greenhouse infrastructure could create a sustainable, low-labor food source for children in vulnerable communities. Additionally, this approach supports the foundational recommendation of maintaining a healthy diet as part of climate-resilient health strategies.

Research gaps and future directions

A significant longitudinal and methodological gap exists in the literature linking climate change to pediatric dermatologic outcomes. Most available studies are cross-sectional or event-driven, which offer limited insights rather than capturing long-term trends. This gap is particularly pronounced in geographic regions where children are most susceptible to climate-related stressors such as extreme heat, drought, flooding, and wildfires. The American Dermatological Association, in its recent climate change statement, underscored the urgency of expanding research efforts, including the implementation of novel diagnostic coding systems to more accurately identify and track skin conditions associated with environmental change [10]. Moreover, current research in environmental dermatology inadequately represents the populations most at risk. Children with skin of color, as well as those from socioeconomically disadvantaged or climate-vulnerable communities, remain underrepresented in existing datasets [11]. This underrepresentation limits the ability to apply findings broadly and poses a direct clinical risk: the clinical morphology of many dermatoses in skin of color, particularly in pediatric patients, can differ significantly from classic descriptions. For instance, AD, a condition exacerbated by climate-related environmental factors, may present as violaceous or ashen gray plaques in children with skin of color rather than the classic erythematous rash described in textbooks, leading to potential misdiagnosis [32]. This discrepancy contributes to delayed treatment and an incomplete understanding of the true prevalence of climate-related skin diseases in diverse populations. Given that dermatologic responses to environmental stressors are modulated by factors such as baseline skin pigmentation, genetic predisposition, and environmental exposure, future research must adopt a geographically diverse and longitudinal approach to fully understand the pediatric dermatologic consequences of a changing climate. 

To address critical data deficits and facilitate the early detection of climate-related dermatologic conditions in children, a surveillance infrastructure for pediatric dermatology is urgently needed. This infrastructure should integrate real-time dermatologic data with environmental indicators through dual monitoring systems. The recent recommendation to develop new International Classification of Diseases (ICD) codes dedicated to climate-related skin conditions represents an advancement, as such codes would standardize data collection and allow aggregation of cases across regions, prioritizing data from high-risk, climate-vulnerable, and underrepresented areas to improve global representation. This system will fill a critical infrastructure gap and provide an evidence base for clinical and public health interventions. 

Additionally, a significant opportunity lies in education reform to prepare future healthcare providers for the consequences of climate change. Medical school and residency programs should develop and mandate curricula on climate change and health, with specific modules on dermatologic impacts. Equally important is the inclusion of training on health equity and how climate change may disproportionately burden certain pediatric populations. Encouragingly, survey data reveal that the majority of pediatric trainees and program directors support the integration of climate-health education into medical training [33]. The American Dermatological Association has also recommended expanding climate-health instruction as part of its formal policy response to climate change [10]. Institutionalizing climate-health literacy within medical education will equip pediatricians and dermatologists not only to recognize environmental contributors to skin disease and counsel patients effectively, but also to engage in broader advocacy efforts.

## Conclusions

Climate change is increasingly affecting pediatric skin health, yet key gaps remain in research, education, and policy. Children, especially those with skin of color or in high-risk regions, face disproportionate risks from environmental stressors. Addressing these challenges requires longitudinal research, better representation, climate-focused medical training, real-time surveillance, and policy integration. Protecting pediatric skin health must be a core component of larger climate resilience efforts to ensure equitable care for all children. 
